# Clinical experiences of staff and students in transitioning from in-person to blended teaching

**DOI:** 10.3389/froh.2024.1306421

**Published:** 2024-03-11

**Authors:** Melanie Nasseripour, Ana Angelova Volponi, Susha Rajadurai, Jonathan Turner, Muna Dahir Hassan, Anitha Bartlett, Jonathan San Diego

**Affiliations:** ^1^ Centre for Dental Education, Faculty of Dentistry, Oral and Cranio-Facial Sciences, King’s College London, London, United Kingdom; ^2^ Faculty of Nursing, Midwifery & Palliative Care, King’s College London, London, United Kingdom

**Keywords:** blended learning, narrative research, thematic analysis, online learning, teaching modalities, clinical teaching

## Abstract

This paper describes some of the lessons learned during the COVID-19 pandemic from a study conducted with a group of clinical teachers and undergraduate dental students at the Faculty of Dentistry, Oral & Craniofacial Sciences (FoDOCS) at King's College London about the use of a combination of remote, online and in-person teaching methods that resumed from June 2020. In the narrative research, participants shared their experiences delivering online clinical workshops and their previous experiences delivering face-to-face sessions online, both during and before the pandemic. We conducted remote interviews with the participants via video conferencing, which were recorded, transcribed, and analysed using thematic analysis. Narrative accounts revealed commonalities organised into seven themes, highlighting some of the challenges encountered during the pandemic and providing insights into addressing different curricular constraints and concerns when utilising various delivery modes during emergency situations, such as pandemics. In our study, we concluded that students and teachers benefit from dissociating clinical learning from clinical treatment sessions to focus on the educational intent and content before applying them chairside with patients. Throughout the course, students and teachers were challenged by a lack of engagement. In addition, it is important to examine the online fatigue highlighted by both students and teachers and identify ways to improve time, literacy, and facilitation to create a more conducive learning environment.

## 1 Introduction and background

During the pandemic, higher education institutions embraced innovative pedagogical approaches that involved technology-enhanced learning (TEL). Blended learning (a combination of in-person and online learning) became part of mainstream teaching and learning as it supports the use of TEL in teaching session activities involving training, presentations, and discussion groups in both synchronous and asynchronous modes (1). Alammary (2) suggested five components of blended learning, which combine face-to-face (1) teacher-led instructions and (2) collaboration among students on specific learning activities with online (3) teacher-supervised instructions, (4) collaborative student work in an online environment, and (5) unsupervised self-paced student activities. Several factors influence the effectiveness of blended learning approaches (1–3). Some of the factors are categorised based on their relation to students, teachers, and technologies (3).

Blended learning has its strengths and limitations. Several studies suggest that blended learning positively affects the learning process, assessment, and outcomes (1–4). However, during the pandemic, the hybrid mode of in-person and online teaching and learning introduced challenges, barriers, and limitations (5, 6). Blended learning can provide students with more flexibility in accessing and interacting with learning materials, allowing them to learn at their own pace. However, the lack of face-to-face interaction can make it difficult for teachers to provide personalised feedback and guidance to students, which can lead to a lack of engagement and motivation. In addition, the difficulty of managing and monitoring online activities of students can present a challenge for teachers.

Narrative accounts from students and staff can provide insight into the lessons learned from introducing blended learning during the COVID-19 pandemic. A number of dental institutions, like other disciplines in higher education, were compelled to adopt and establish online delivery of education. Providing in-person training, in-person learning, and supervised teaching in clinical settings is a well-established pedagogical approach that has long been in place. Therefore, the sudden emergence of a pandemic presented a significant challenge for dental education institutions.

Undergraduate clinical students attending clinical courses and training at our Faculty of Dentistry, Oral & Craniofacial Sciences (FoDOCS) at King's College London did not experience prolonged periods of remote teaching and training.

The teaching and learning activities, which ran within the clinical teaching sessions and their impact on the quality of learning outcomes achieved, encompassed:

• Synchronous discussions in person and online clinical case-based scenarios.
• Asynchronous discussions on posting clinical specific questions facilitated by clinical teachers to moderate the discussion/postings from students.
• Synchronous video conferencing seminars centred around a specific clinical case scenario, with breakout sessions for students to look at the case together and be back in the seminar, followed by moderate discussions with clinical teachers present.

Moreover, staff and students alike have experienced challenges in transforming teaching into a blended mode during the pandemic. Given the constraints of safe distancing and the changing traditional teaching practices to the online environment, clinical teachers had to provide clinical care to patients while also training students.

In response to finding an alternative to our teacher-centred (face-to-face) clinical education model and ensuring continuous development of clinical knowledge, reasoning, and skills, we explored alternative teaching approaches. Learning and teaching resources in the virtual learning environment (VLE) were rapidly redesigned, and video conferencing tools were adopted and applied in teaching clinical sessions for the clinical training and teaching of undergraduate dental students. Online bulletin boards were used to post clinical scenarios on asynchronous forums with polling, informed by problem-based learning. This platform enabled students to learn, discuss, and debate with peers online regarding the management of a clinical problem using the latest evidence facilitated by clinical teachers.

Facilitated synchronous communications in debrief seminars were conducted via MS Teams meeting chat rooms (e.g., problem-based learning chat rooms).

Narrated PowerPoint lectures, lecture capture videos, and recordings of the debrief seminars were made available for access 24 h a day.

The purpose of the present paper was to examine the clinical teaching practices involved in blended learning by analysing student and teacher narratives. Narrative research [see (7)] was conducted to investigate the lived experiences of a small group of teachers and students engaged in blended, in-person, and remote teaching practices.

## 2 Methods

The research aims to explore and provide insight into the lived experience of students and staff at FoDOCS during the Covid-19 pandemic, especially regarding the rapid introduction and implementation of technology-enhanced learning.

The study has been approved by the KCL Research Ethics Committee (LRS-20/21-20813, PNM Research Ethics Panel).

### 2.1 Researchers, participants, and settings

The staff participants were recruited via purposive sampling from the group of clinical teachers who teach in our Undergraduate Integrated Clinical Care clinics in the restorative disciplines. These teachers have been involved in delivering online clinically themed workshops and had previous experience delivering these face-to-face sessions. Student participants comprised undergraduate Year 4 (BDS4) students from the 2020/21 cohort who attended the sessions online during the pandemic and had previous experience of attending clinical sessions delivered face-to-face. The strategy and approach to online delivery of clinical teaching for the BDS4 cohort was representative of the strategy adopted for all clinical teaching sessions for all clinically active dental students, i.e., BDS2/3/4/5. All participants volunteered to take part in the study. Each participant was sent a detailed study information sheet and given a minimum of 24 h to decide on participating in the study.

**Figure 1:**
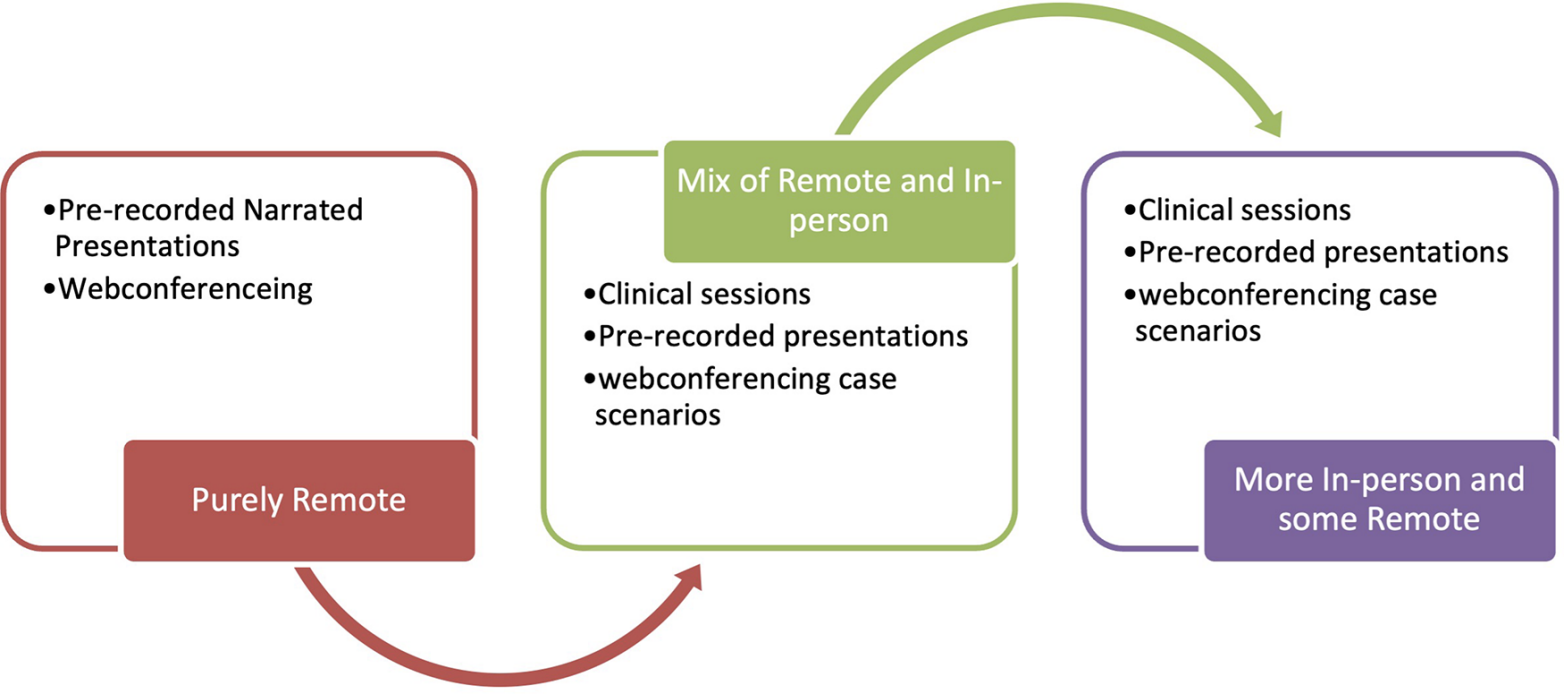
Technologies and modalities used at the Faculty of Dentistry, Oral & Craniofacial Sciences, King’s College London during the pandemic: mix of remote, online, and in-person sessions.

The FoDOCS curriculum was very specific in its approach during the pandemic, outlined as follows (Figure 1):

• From March 2020 to June 2020, the dental undergraduate curriculum was delivered fully online and focused on theories relevant to familiarising and equipping students with the knowledge and skills required for different clinical situations they may encounter traditionally (purely remote).
• From June 2020, a hybrid curriculum was implemented, with some in-person clinical sessions, mainly consultant clinics and outreach patient care, as well as simulation clinics in lieu of patient care at our main teaching hospitals (a mix of remote and in-person).
• As on-campus clinical teaching and learning were prioritised for BDS5 (final-year students as they graduate within 3 or 4 months), the BDS4 cohort continued with a combination of some on-campus simulation activities and asynchronous/synchronous online teaching and learning (more in-person and some remote).

### 2.2 Interview and identification of narrative accounts

Participants were asked questions and prompted to narrate the events that transpired relating to the questions, as recalled by one of the researchers (Table 1). Initially, general questions were asked to gain a better understanding of their teaching experiences. These were followed by specific questions related to teaching and learning practices.

**Table 1:** Interview questions and prompts for students and teachers at the Faculty of Dentistry, Oral & Craniofacial Sciences, King's College London.

Staff (clinical educators)	Students
How do you feel about online teaching being more embedded in the curriculum? Sum up the pros and cons regarding online clinical teaching. (What went well and what did not go so well?) Do you feel that your students’ clinical competency (or knowledge) has improved following the online clinical teaching sessions?	How do you feel about online teaching being more embedded in the curriculum? Sum up the pros and cons regarding online clinical teaching. (What went well and what did not go so well?) Do you feel that your clinical competency (or knowledge) has improved following the online clinical teaching sessions?
How did you experience your role as a clinical teacher during the online teaching sessions? React to the statement: “It was easy for me to interact with my students during the online clinical sessions.” What are your positive and negative experiences in online clinical teaching sessions with case-based scenarios? (What went well and what did not go so well?) Do you feel the learning outcomes set in the module (programme) have been delivered? If not, can you identify the ones that were not? What has been your experience with posting of clinical specific questions on an online forum	What has been your experience with online MS Teams seminars around a specific clinical case scenario? React to the statement: “It was easy for me to interact with my teachers during the online clinical sessions.” What are your positive and negative experiences in online clinical teaching sessions with case-based scenarios? (What went well and what did not go so well?). Do you feel the learning outcomes set in the module (programme) have been delivered? If not, can you identify the ones that were not? What has been your experience with posting of clinical specific questions on an online forum.
Did you feel students were engaged in the online teaching sessions? React to the statement: “During online sessions, I had more time to discuss and reflect on different clinical aspects with my students.” React to the statement: “During online clinical teaching sessions, students worked more collaboratively and have boosted their team working skills.”	Do you feel that you were engaged in learning during these sessions? React to the statement: “During these sessions, I had more time to discuss and reflect on different clinical aspects with my clinical teachers.” React to the statement: “During online clinical teaching sessions, I worked more collaboratively and have boosted my team working skills.”

**Figure 2:**
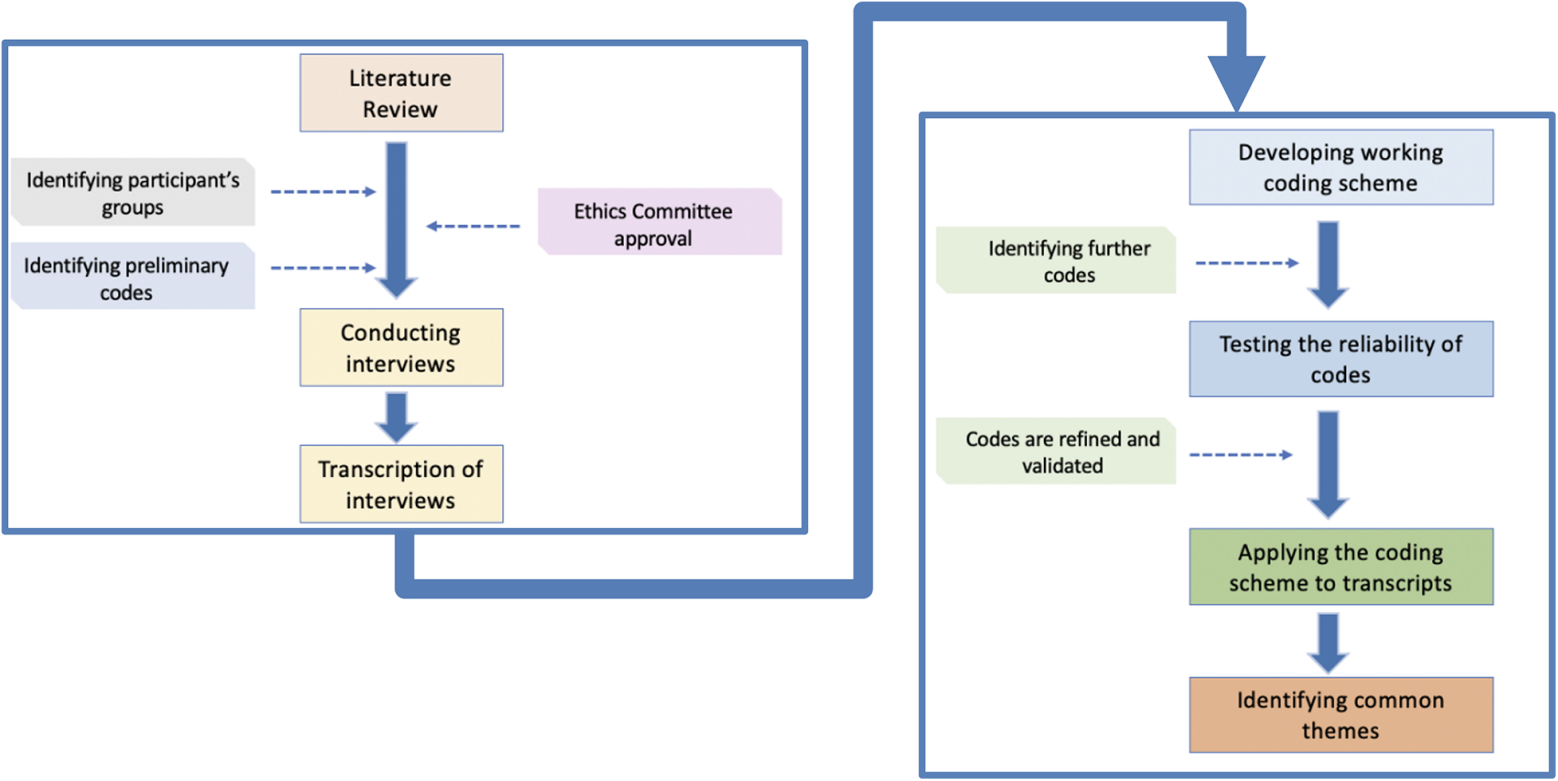
Interview conduct and analysis of narrative accounts of students and teachers at the Faculty of Dentistry, Oral & Craniofacial Sciences, King’s College London.

Interviews were conducted by researchers who were not directly involved with teaching the students or working with the clinical teachers. The sessions were recorded, and narrative accounts were transcribed using automatic captioning. Interviewers reviewed and analysed the transcriptions. No video images were recorded. The overall process is summarised in Figure 2.

### 2.3 Analysing narrative accounts: thematic analysis

In analysing narrative data, thematic analysis was used to identify commonalities and differences in the ideas and phrases that students and teachers articulated in their narratives and that can indicate some degree of importance allocated to a specific thought or occurrence. This research used three aspects of identifying themes (7):

• Recurrence criterion, referring to concepts that are repeated using similar words or phrases,
• Repetition criterion, meaning that an idea is conveyed with the use of the same words,
• Forcefulness, referring to the emphasis applied to a concept.

**Figure 3:**
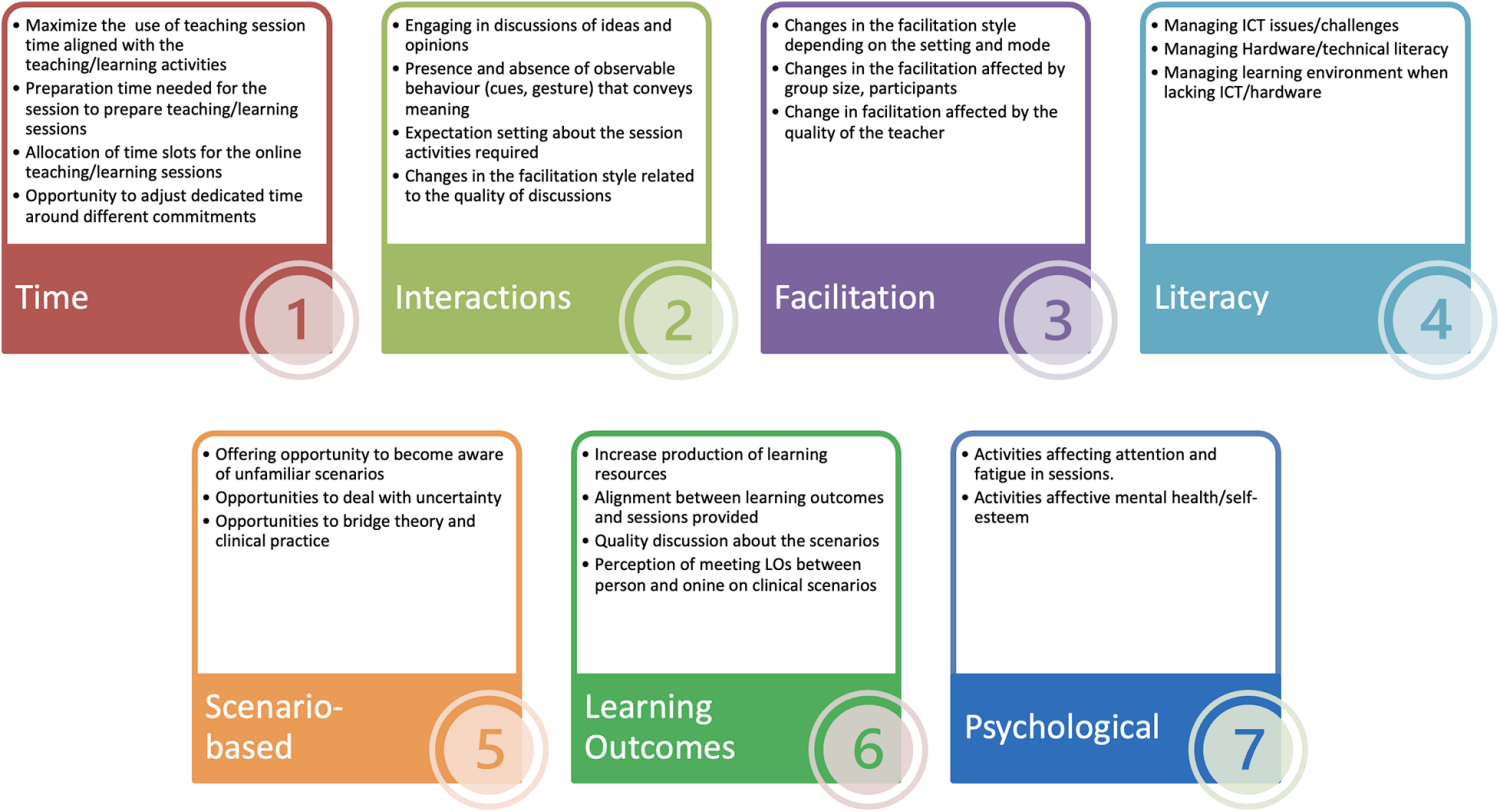
Identified commonalities and thematic categorisation for coding of narrative responses regarding online education during the COVID-19 pandemic by a sample of students and teachers at the Faculty of Dentistry, Oral & Craniofacial Sciences, King's College London.

The coding process (Figure 3) stemmed from an inductive approach, and the themes were progressively refined (data familiarisation, initial coding, and generating themes from the coding). They described the perceptions of the participants as interpreted by the researcher, who became a “storyteller who interpreting data through the lens of their own cultural membership” (8) in this context of oral health education at FoDOCS (King's College London).

Following the transcription of each interview, the transcripts were reviewed by multiple members of the research team to highlight and note the major salient themes. Narrative responses were coded and then reanalysed for commonalities, which were then used to identify the themes. The themes were communicated to the research team, and at a consensus meeting, the themes were discussed, peer-validated, and agreed upon. A coding scheme with the description was made available to two researchers calibrated to code the transcripts.

## 3 Results

The narrative accounts were derived from responses during the interviews, which represented lived experience of Year 4 Bachelor of Dental Surgery programme students (*n* = 3) and clinical teachers (*n* = 5) teaching in our Undergraduate Integrated Clinical Care clinics and supervising in the restorative discipline within the context and setting at King's, as presented in Section 2.1.

The implementation of a mix of fully online, hybrid, synchronous, and asynchronous facilitation of teaching presented pedagogical challenges and constraints that may impact the subjective views of the narrative. However, reflexivity in analysing the accounts considered different factors that may unavoidably be featured by the participants in presenting their experiences.

Commonalities identified in the coded narrative accounts were reanalysed and scrutinised by the research team after the researchers validated the coded narrative accounts. Seven themes were agreed as the main findings.

Short descriptions of the commonalities within each of the seven themes are presented in Figure 3.

Table 2 shows examples of narrative accounts. A total of 26 sub-themes were identified, each give a distinct brief description as labels. Numeric coding is for ease of use in referring to the themes and for the purposes of researchers’ coding scheme operationalisation.

**Table 2:** Description of identified commonalities by themes and corresponding examples of quoted narrative responses regarding online education during the COVID-19 pandemic by a sample of students and teachers at the Faculty of Dentistry, Oral & Craniofacial Sciences, King's College London.

Theme	Descriptions of sub-theme commonalities	Example of quoted narrative responses
1	Time: maximise the use of teaching session time aligned with the teaching/learning activities	*So now we've got a pure hour to go through the tutorials* (Teacher 1)
1	Time: preparation time needed for the session to prepare teaching/learning sessions	*With online like for example with the lectures that we had a pre recorded that means that you can do them at your own pace. So you kind of control the rate of like how you'll study ‘cause when we had lectures face to face it was really difficult to attend every single lecture or like don't even attend like you would go there then* (Student 2)
1	Time: allocation of time slots for the online teaching/learning sessions	*I think if you are going to embed days or even weeks of online teaching then they need to be timetabled some downtime or in-between those sessions* (Teacher 5)
1	Time: opportunity to adjust dedicated time around different commitments	*So it also gives flexibility to students and teachers* (Teacher 5)
2	Interactions: engaging in discussions of ideas and opinions	*Then I think, yeah, I think that’s just because we had our cameras off so we wouldn't be as engaged because face to face, your teacher can see you. They can see what you're doing. They can see if you're talking to someone else, so you have to stay focused. UM, but online. There's no accountability. Nobody can see what you're doing so. Yeah, you can kind of lose engagement* (Student 2
2	Interactions: presence and absence of observable behaviour (cues, gestures) that conveys meaning	*They are following what you're saying by nodding their head yes. With the body language* (Teacher 5)
2	Interactions: expectation setting about the session activities required	*I told them that in advance that I've got your names, they're going to be asking you questions. And I expect you to answer. And that way it kept their attention and they knew there had to be there* (Teacher 4)
3	Facilitation: changes in the facilitation style related to the quality of discussions	*Unfortunately, it becomes a monologue in a bit like Now I'm talking to you and I can't see you…You know, it’s the detached voice* (Teacher 4) *There are times when you're kind of waiting for some interactions with students, nothing is forthcoming* (Teacher 3)
3	Facilitation: changes in the facilitation style depending on the setting and mode	*You tend to find some students interact very well… but then again that was probably not dissimilar to, to face-to-face tutorial teaching. You'll find some of the students who, who give a lot back* (Teacher 3)
3	Facilitation: changes in the facilitation affected by the group size of participants	
3	Facilitation: change in facilitation affected by the quality of the teacher	*You have different teachers, different ideas, different delivery, different experience. And the thing is the main student complaint was What was being given to them was different ideas, different delivery, different experience. And the thing is the main student complaint was What was being given to them was different.* (Teacher 2)
4	Literacy: managing ICT issues/challenges	*I think are not used to teaching online. UM, so they found it quite hard to adapt at the beginning, so that impacted the learning that was provided to us kind of cause and they wouldn't be able to explain things. Online because they would want to be showing us models or like uhm. Or like a textbook paid or something, and they wouldn't know how to configure that and they wouldn't know how to share their screen either. So sometimes they had examples where they didn't know how to share screen. And so I think. I think they are trying their best, but maybe like training on technology might be useful* (Student 1)
4	Literacy: managing hardware/technical literacy	*Seminar where the IT that wouldn't connect. And now IT at King’s has always been poor* (Teacher 2)
4	Literacy: managing the learning environment when lacking ICT/hardware	*I think that it was a very steep learning curve …* (Teacher 4)
5	Scenario-based: offering opportunities to become aware of unfamiliar scenarios	
5	Scenario-based: opportunities to deal with uncertainty	*No way to … connect theoretical side and say their clinical practices* (Teacher 4)
5	Scenario-based: opportunities to bridge theory and clinical practice	
5	Scenario-based: opportunities to develop teaching practice	*Now we have lectures, online on our Keats space and they're all narrated and everything, and looked at them and ever, so that’s really good* (Student 1)
6	Learning outcomes: increase the production of learning resources	*We are actually generating a lot more material now, resources and updating the resource is because we're moving it online* (Teacher 1)
6	Learning outcomes: alignment between learning outcomes and sessions provided	*We've made sure that those tutorial sessions are delivering certainly what we intended before and probably more so because of where we are* (Teacher 1)
6	Learning outcomes: quality discussion about the scenarios	*Actually, go into a lot more depth with these cases.* (Teacher 1)
6	Learning outcomes: perception of meeting LOs between in-person and online clinical scenarios	
7	Psychological: activities affecting attention and fatigue in sessions	*But sometimes those classes would overrun so you would just be sitting down for three hours and it would be really hard to concentrate. So you just started to get a bit fatigued and loose your concentration faster, but I guess yeah.* (Student 2)
7	Psychological: activities affecting mental health/self-esteem	*And the biggest problem for students is asking questions. A lot of them don't want to be really ridiculed* (Teacher 2)

## 4 Discussion

The pandemic has presented several pedagogical challenges and curricular constraints. Due to the large size of our student population at King's FoDOCS in the United Kingdom, our experience in providing clinical education has enabled us to offer a combination of fully online, blended, and in-person teaching within clinical settings, as well as virtual and face-to-face tutorials.

We may not be the only dental faculty to experience this. Hence, higher education institutions need to be aware of the narrative accounts that provide insight into the different practices encountered and how they were addressed. The mix of delivery modes has created new challenges for clinical teaching and learning. Based on the qualitative analysis of the seven identified themes in student and teacher lived experiences, we offer suggestions for dealing with curricular and pedagogical constraints in clinical teaching and learning. The challenges may therefore be managed more structurally, systematically, and innovatively in the event of a similar pandemic situation.

### 4.1 Time

The narrative highlighted Time as a theme relating to constraints on time spent in sessions, timing or timetabling, and time management. Participants relate this notion of time to the efficiency, effectiveness, and appropriateness of teaching and learning activities. For instance, several narrative comments alluded to the flexibility offered by mixed delivery methods and even opportunities that allowed for the theoretical aspects of the curriculum to be delivered online, making student rotations for clinical scheduling easy. This confirms experiences reported by other institutional and healthcare students (9). The below narrative comments somehow confirm views shared by both students and staff:

*”They've put the teachers on a timetable so we are not consistently with a group…ended up teaching groups that…would never normally teach and wouldn't normally see on the clinic”. (Teacher Participant 4)*

*“… Definitely did have more time. … it*’*s always busy on clinics that you don't really have that much time to discuss through a patient in full detail …. So it*’*s a bit more relaxed, a bit more time and then you can discuss at your own pace, we definitely did have more time. (Student Participant 1)*

*“We spent most of the time looking for student … in a very limited amounts of time. Whereas online, you know that that*’*s what they're doing for the afternoon or morning.” (Teacher Participant 4)*.

Staff brought up timetabling as both a positive and negative aspect of online teaching. While some saw the online delivery as a better use of the timetable, others felt challenges in managing session scheduling. However, the accounts seemingly suggest that both students and staff were less pressured by time constraints as scheduling provided more flexibility (9). The narratives highlight the productive use of session time and fewer preparation requirements to manage clinical activities and teaching and learning activities between mixed modes of session delivery.

### 4.2 Interaction

Goetz et al. (10) reported that students missed contact with other students. Indeed, our students commented on the difficulty in connecting with others. Some were reluctant to ask questions, with one highlighting that teachers were unable to read body language online and see when there was confusion and another mentioning that recording the session is a disincentive to asking questions. It seems that being able to turn cameras off also promoted disengagement. Wang et al. (11) similarly reported that the interaction between teachers and students showed the lowest satisfaction in an online teaching environment:

*“I think it*’*s the online learning ‘cause sometimes these are people that would be asking questions in class, UM, but for some reason they wouldn't online.” (Student Participant 2)*

*“the biggest problem with this online, specially recording it, people are too scared to broach their views” (Teacher Participant 2)*

and that perhaps “*the biggest problem for students is asking questions. A lot of them don't want to be really ridiculed.” (Teacher Participant 2)*.

“The elephant in the room” in terms of engagement is, of course, the fact that it is one thing to sign into an online session but quite a different thing to be engaged, as Aivaz and Teodorescu (12) reported increased student distractions and multitasking within the virtual learning environment:

*“they can check in at the beginning of the seminar, turn the camera off, go do something else and turn it back on at the end so collaboratively as they did before in the way we used to do the face-to-face session”. (Teacher Participant 4)*.

*“I had two of the students that interacted. The rest they may not have as well have been there there*’*s very little interaction.” (Teacher Participant 2)*

*“the interaction with the students is actually reduced.” (Teacher Participant 2)*

*”that*’*s just because we had our cameras off so we wouldn't be as engaged because face-to-face, your teacher can see you. They can see what you're doing. They can see if you're talking to someone else, so you have to stay focused.” (Student Participant 2)*

Even though some staff felt that on-campus (in-person) sessions allowed for a better read of the audience, with the ability to make eye contact with moving about the room and using the space, there is also an argument for considering that student behaviour was no different from that in seminar rooms, with committed students in the front row and disengaged ones in the back. Nevertheless, on-campus rooms were often not equipped for students to take notes and access the resources simultaneously during the seminar.

*“face-to-face tutorials ……  you can sort of make eye contact with those students who aren't necessarily participating” (Teacher Participant 1) and that “you can struggle sometimes actually knowing who*’*s there … you can't see a face you can only see an initial.” (Teacher Participant 1)*

*“some students interact very well … but then again that was probably not dissimilar to face-to-face tutorial teaching.” (Teacher Participant 3)*

This was in accordance with “improved accessibility” being considered as one of the benefits of online dental education, as reported by Kerkstra et al. (13). The fact that the synchronous tutorials were recorded allowed students to revisit the session at a later point, which potentially had a negative impact on attendance and engagement during a session:

*“help that sessions are recorded because they know that people are gonna hear them later.” (Student Participant 2)*

*“So it*’*s a bit more relaxed, a bit more time and then you can discuss at your own pace.” (Student Participant 1)*

The challenge remains in an online environment to properly gauge the engagement or even the presence or absence of the students within the tutorial. This issue with students exhibiting a lack of interest and motivation, with increased issues of absenteeism and distraction during online classes, was reported by Iqbal et al. (14).

Whether in an online or on-campus setting, the challenge to connect and engage with the audience remains and is also related to the facilitator and their skills (clinical and pedagogical) in maintaining a captivated audience.

### 4.3 Facilitation

When looking at facilitation, we can see an intuitive link with the interaction theme, and these aspects mutually impact each other. However, here, the participants are outlining very defined issues concerning teaching and learning styles related to synchronous/asynchronous, online, and on-campus modalities:

• Smaller group sizes in the online environment appeared more conducive to group discussions, as there was a general reluctance from many students to ask questions. This observation is also reported by Kaczmarek et al. (15) as improving student engagement and understanding:
  *“so that*’*s been really useful, especially when you have tutors which are like engaging and getting you a bit more involved as well.” (Student Participant 1)*
• Ability to ask a question in the chat function on Teams, however, rather than verbally asking:
  *“If I have a question though I do always just ask it and that*’*s no different to being face-to-face, but I think that*’*s just me.” (Student Participant 2)*
• Anonymised chat function may help overcome reluctance to speak in the online sessions:
  *“sometimes these are people that would be asking questions in class, UM, but for some reason they wouldn't online … don't know what changes?” (Student Participant 2)*
  *“We need to, we need to anonymize the chat line basically so that they can ask questions most of them are so scared.” (Teacher Participant 2)*
  *“it doesn't help that sessions are recorded because they know that people are gonna hear them later.” (Student Participant 2)*
• Being alone at home rather than with colleagues created a barrier regarding group or collaborative work:
  *“there have been other classes where somebody is asked a question and then you've bounced ideas off each other, but it*’*s only really two people. They're speaking like two students and a teacher, and so I don't think the group discussion happened so much.” (Student Participant 2)*

The difficulty for staff was finding the right approach to online teaching, as their point of reference was in-person teaching. For some, it was related to their speaking, teaching style, and ability to use body language and read body language:

*“ face to face, then you, It*’*s more a personable experience because you can see the entire person” (Teacher Participant 5)*

*“I use my hands and my body, my body movement and be like the conductor in an orchestra. You can't do that online. Yeah. So you know, So you know, teaching is often putting on a performance, isn't it?” (Teacher Participant 4)*

Nonetheless, adjustments were made and staff found ways to make the online environment work for them using synchronous and asynchronous formats to support student learning:

*“you can share PowerPoint presentations quite easily. You can share articles; you could show them pictures. There*’*s even a blackboard thing.” (Teacher Participant 5)*

*“… it would be difficult to make notes. Whereas with pre-recorded lectures you can go to the point that you wanted like a like.” (Student Participant 2)*

*“Play back basically.” (Student Participant 2)*

### 4.4 Literacy

Concerns regarding literacy, including digital literacy and pedagogical literacy, are essential to address as the narrative seemingly shows that even with a return to on-campus activities, there are benefits to keeping the mix of synchronous, asynchronous, online, and on-campus modes. The affordance offered by the technology and ability of a teacher to share their screen enabled resources such as photographs to be viewed more clearly by students; however, conversely, teachers were unable to pass around physical objects such as models in the online environment. However, with this technological advancement, similarly to Kumar and Vigil (16), students highlighted that staff had developmental needs that would require support, such as how to fully use the technology—such as sharing documents online. Indeed, the online teaching process requires support in technological and pedagogical aspects, including tools, resources, and training courses, as echoed by others (17). This convenience and comfort of learning from anywhere at any time is similarly reported by others (18). There were narratives that also highlighted the ability to have an individualised pace of learning, which was found by others (19). There was also evidence that the lack of good internet access, adequate place for online teaching, difficulties in producing teaching materials, and home/personal life commitments had a significant impact on the quality of life and anxiety scores for teachers (20):

*“people have a lot of Wi-Fi problems. So if they can't access a tutorial because of their Wi-Fi problems.” (Student Participant 2)*

*“technical problems. It*’*s not always that easy to get, good Internet.” (Teacher Participant 3)*

*“So if they can't access a tutorial because of their Wi-Fi problems” one student points out. (Student Participant 2)*

*“they found it quite hard to adapt at the beginning they wouldn't know how to share their screen either.” (Student Participant 2)*

*“they didn't know how to share screen. And so I think. I think they are trying their best, but maybe like training on technology might be useful.” (Student Participant 2)*

*“it was so fairly daunting to start off with because you're unfamiliar with the technology.” (Teacher Participant 1)*

*“haven't totally embraced all the features of teams about sort of using breakout groups.” (Teacher Participant 1)*

*“If we had adequate sort of office space, from which to work, then we could deliver online teaching to students who aren't in the building, whilst being physically in the building ourselves to go on and do clinical teaching later on.” (Teacher Participant 3)*

*“They felt frustrated and had to use any available means for example when on campus when I did the online teaching and I was using my phone because the facilities are not there.” (Teacher Participant 2)*

Both students and staff highlighted concerns and anxiety around connectivity issues, as they proved to be a barrier for some in accessing the sessions. Students also could see the staff struggling with the technology and reported highlighting developmental needs in technology usage in teaching. Interestingly, staff also highlighted technical issues, lack of access to adequate resources, and the need for more support and training.

The accounts also made apparent suggestions to provide appropriate resources on campus to enable the delivery of online tutorials—such as quiet office space with computer access. It was also noted that some students were accessing sessions using mobile phones and tablets rather than computers, which potentially impacted their experience. However, this issue is related to on-campus challenges. The availability of recordings, which are a technology affordance, was deemed much more useful in case students encounter issues attending in person at the time of the tutorial.

### 4.5 Learning outcomes

The accounts represented a mix of views about how learning outcomes are met and how these are met in different delivery modalities. The results confirm unfavourable views around the acquisition of clinical skills in an online teaching environment, which was similar to previous empirical research presented by others (21, 22). There were concerns about the mix of delivery methods and the disruption, leading inevitably to skill deficits within the cohort of students’ experiences during the pandemic (23). Previous research suggests concerns about online learning not being the best way of communication, especially in medical and dental programmes. The narrative accounts presented comments:

*“It might sometimes occur bit off topic, and then you still discussing dentistry and stuff, but just a bit not what was on the kind of written for that tutorial. So yeah, but we do cover most of the outcomes and stuff.” (Student Participant 1)*

*“We are actually generating a lot more material now, resources and updating the resource is because we're moving it online” (Teacher Participant 1) and “We've made sure that those tutorial sessions are delivering certainly what we intended before and probably more so because of where we are.” (Teacher Participant 1)*

*“Without the Face-to-face clinical teaching, inevitably, they haven't gained as much of a grasp of the clinical things.” (Teacher Participant 3)*

*“the practical bits are less, less easy to grasp virtually or online.” (Teacher Participant 3)*

*“don't understand about independent self-regulated learning” (Teacher Participant 5) and “I think it*’*s about them not taking accountability for their learning.” (Teacher Participant 5)*

In terms of learning outcomes, students felt that some were readily met in the online environment, such as specific tutorial topics. Their confidence in approaching specific clinical situations was increased. As echoed in Jabbour and Tran (24), students mentioned that the sessions were useful, however, the students highlighted that these sessions do not substitute clinical experience. On the other hand, staff felt that delivering academic content and associated learning outcomes was achieved satisfactorily, with a great volume of online resources generated for student use. Interestingly, more care seems to be put into online teaching because it must also cover more than the clinical experience. However, there was a concern about the inability to teach the hands-on clinical material or see whether the online teaching impacted the students’ clinical activity. Among clinical aspects, the staff highlighted communication skills and the need to observe interactions with patients to give feedback to students regarding their communication skills. There were accounts that seemingly suggest that the students lacked maturity and the ability to manage their own learning, which is fundamental to any online delivery of the programme.

### 4.6 Scenario-based and psychological

With regard to the period following an online teaching session, although the main concern was the potential impact on future clinical activity due to the lack of hands-on experience, as detailed above, there were also questions about the impact on student and staff wellbeing, mentioning the following:

• Some sessions were very long, where 3-h clinic sessions turned into 3-h online equivalent sessions with case studies.
• Tutorials had to be all placed into an online day.

This meant that students would have back-to-back online seminars in different disciplines and teachers would have back-to-back seminars for different year groups. A novel issue raised by students was what we have categorised as “online fatigue”. Difficulties arising from sitting in front of a screen for long periods of time also lead to a loss of concentration:

*“sometimes those classes would overrun so you would just be sitting down for three hours and it would be really hard to concentrate. So, you just started to get a bit fatigued and lose your concentration faster.” (Student Participant 2)*

*“quite, quite tiring, online teaching all day, I mean, I think it must be quite tiring online learning.” (Teacher Participant 3)*

*“to be timetabled some downtime or in-between those sessions” and “for them to just reflect or just to get a cup of tea and stretch their legs.” (Teacher Participant 3)*

*“cameras off and the teacher can't see us, so we're kind of fidgety. And we're not paying attention.” (Student Participant 2)*

Some staff also pointed out the inability to interact on a more social level with students in the online environment. They would not have “*the social interaction and downtime between lessons*” *(Teacher Participant J3)*.

The study has limitations. It presents narrative accounts from a small number of participants. However, the recurring themes represent saturation within the presented narratives. It is also important to consider and account for the specific needs of different year groups. The time commitment presented a challenge for many who expressed interest in participating in the study.

## 5 Conclusion

The approach we adopted in this study provided researchers with the opportunity to explore participants’ perspectives and experiences and identify potential areas for further research. In addition, it allowed researchers to gain insight into the challenges and opportunities that teachers encounter in their classrooms.

In both teacher and learner populations, we observed significant concerns regarding engagement, not limited to attendance, connection, and interaction, and we noted an emerging concept of online fatigue. Concerns like these can undermine the teaching and learning process.

To help learners and teachers focus on the educational intent and content, clinical learning should be separated from clinical sessions. Simulations and virtual patients can be used to establish clinical learning outcomes prior to the expected application chairside. By the time students arrive at clinics, they should be supported, prepared, and scaffolded, regardless of their learning style.

It is important to note that both teaching and learning communities now have alternatives to conventional methods and modalities. Thus, returning to a full-time campus presence would not be feasible. However, to ensure a more conducive learning environment, it is crucial to take stock of online fatigue highlighted by both students and teachers when online sessions were introduced. Further research is recommended to provide evidence-based support for this approach as “improvement.”

## Data Availability

The raw data supporting the conclusions of this article will be made available by the authors without undue reservation.

## References

[1] Kintu MJ Zhu C Kagambe E. Blended learning effectiveness: the relationship between student characteristics, design features and outcomes. Int J Educ Technol Higher Educ. (2017) 14(1):7. 10.1186/s41239-017-0043-4

[2] Alammary A. Blended learning models for introductory programming courses: a systematic review. PLoS One. (2019) 14(9):e0221765. 10.1371/journal.pone.0221765 31487316 PMC6728070

[3] Tong DH Uyen BP Ngan LK. The effectiveness of blended learning on students’ academic achievement, self-study skills and learning attitudes: a quasi-experiment study in teaching the conventions for coordinates in the plane. Heliyon. (2022) 8(12):e12657. 10.1016/j.heliyon.2022.e12657 36643330 PMC9834772

[4] Nasseripour M Turner J Rajadurai S San Diego J Quinn B Bartlett A COVID 19 and dental education: transitioning from a well-established synchronous format and face to face teaching to an asynchronous format of dental clinical teaching and learning. J Med Educ Curric Dev. (2021) 8:238212052199966. 10.1177/2382120521999667 PMC7968006 33796791

[5] Almpanis T Joseph-Richard P. Lecturing from home: exploring academics’ experiences of remote teaching during a pandemic. Int J Educ Res Open. (2022) 3:100133. 10.1016/j.ijedro.2022.100133 36161267 PMC9490561

[6] Littlejohn A Gourlay L Kennedy E Logan K Neumann T Oliver M Moving teaching online: cultural barriers experienced by university teachers during COVID-19. J Interact Media Educ. (2021) 2021(1):1–15. 10.5334/jime.631

[7] Overcash JA. Narrative research: a review of methodology and relevance to clinical practice. Crit Rev Oncol Hematol. (2003) 48(2):179–84. 10.1016/j.critrevonc.2003.04.006 14607381

[8] Braun V Clarke V. Using thematic analysis in psychology. Qual Res Psychol. (2006) 3(2):77–101. 10.1191/1478088706qp063oa

[9] Sarfraz F Daka H Zubair A Sarfraz F. The viability of blended model in undergraduate medical education in COVID-19 pandemic. Pak J Med Health Sci. (2022) 16(1):561–5. 10.53350/pjmhs22161561

[10] Goetz K Wenz H-J Hertrampf K. Certainty in uncertain times: dental education during the COVID-19 pandemic—a qualitative study. Int J Environ Res Public Health. (2023) 20(4):3090. 10.3390/ijerph20043090 36833785 PMC9962035

[11] Wang C Tee M Roy AE Fardin MA Srichokchatchawan W Habib HA The impact of COVID-19 pandemic on physical and mental health of Asians: a study of seven middle-income countries in Asia. PLoS One. (2021) 16(2):e0246824. 10.1371/journal.pone.0246824 33571297 PMC7877638

[12] Aivaz KA Teodorescu D. College students’ distractions from learning caused by multitasking in online vs. face-to-face classes: a case study at a public university in Romania. Int J Environ Res Public Health. (2022) 19(18):11188. 10.3390/ijerph191811188 36141459 PMC9517392

[13] Kerkstra RL Rustagi KA Grimshaw AA Minges KE. Dental education practices during COVID-19: a scoping review. J Dent Educ. (2022) 86(5):546–73. 10.1002/jdd.12849 34978714 PMC9015347

[14] Iqbal FM Lam K Sounderajah V Clarke JM Ashrafian H Darzi A. Characteristics and predictors of acute and chronic post-COVID syndrome: a systematic review and meta-analysis. EClinicalMedicine. (2021) 24(36):100899. 10.1016/j.eclinm.2021.100899 PMC8141371 34036253

[15] Kaczmarek K Chen E Ohyama H. Distance learning in the COVID-19 era: comparison of student and faculty perceptions. J Dent Educ. (2021) 85(Suppl. 1):1197–9. 10.1002/jdd.12469 33070311

[16] Kumar S Vigil K. The net generation as preservice teachers. J Digit Learn Teacher Educ. (2011) 27(4):144–53. 10.1080/21532974.2011.10784671

[17] do Amaral JHL Palmier AC Werneck MAF Lucas SD Senna MIB. Challenges and dilemmas for dental undergraduate teaching with the advent of COVID-19. Pesquisa Brasileira Em Odontopediatria E Clínica Integrada. (2021) 21:e0147. 10.1590/pboci.2021.067

[18] Hertrampf K Wenz HJ Kaduszkiewicz H. Suspension of face-to-face teaching and ad hoc transition to digital learning under COVID-19 conditions—a qualitative study among dental students and lecturers. BMC Med Educ. (2022) 22:257. 10.1186/s12909-022-03335-5 35395749 PMC8992419

[19] Nijakowski K Cieślik K Łaganowski K Gruszczyński D Surdacka A. The impact of the COVID-19 pandemic on the spectrum of performed dental procedures. Int J Environ Res Public Health. (2021) 18(7):3421. 10.3390/ijerph18073421 33806148 PMC8037540

[20] Puccinelli PJ da Costa TS Seffrin A. Reduced level of physical activity during COVID-19 pandemic is associated with depression and anxiety levels: an internet-based survey. BMC Public Health. (2021) 21:425. 10.1186/s12889-021-10470-z 33648487 PMC7919983

[21] Hassan R Khalifa AR Elsewify T Hassan MG. Perceptions of clinical dental students toward online education during the COVID-19 crisis: an Egyptian multicenter cross-sectional survey. Front Psychol. (2022) 12:704179. 10.3389/fpsyg.2021.704179 35069304 PMC8776649

[22] Jum’ah AA Elsalem L Loch C Schwass D Brunton PA. Perception of health and educational risks amongst dental students and educators in the era of COVID-19. Eur J Dent Educ. (2021) 25(3):506–15. 10.1111/eje.12626 33188555 PMC7753269

[23] Nazir MA Khan MR. Exploring the barriers to online learning during the COVID-19 pandemic. A case of Pakistani students from HEIs [higher education institutions]. GIST Educ Learn Res J. (2021) 23:81–106. 10.26817/16925777.1195

[24] Jabbour Z Tran M. Can students develop clinical competency in treatment planning remotely through flipped collaborative case discussion? Eur J Dent Educ. (2023) 27(1):69–77. 10.1111/eje.12778 35103367

